# Prof. C.P. Sawhney: Teacher Par Excellence and a Karmayogi

**DOI:** 10.1055/s-0044-1791984

**Published:** 2024-10-29

**Authors:** Ramesh Kumar Sharma

**Affiliations:** 1 Department of Plastic Surgery, Paras Hospitals, Panchkula, Haryana, India

“Teaching is more than imparting knowledge; it is inspiring change. Learning is more than absorbing facts; it is acquiring understanding.”

—William Arthur Ward

Dr. C.P. Sawhney (popularly known as CPS) tremendously influenced the evolution of plastic surgery in India ( Fig. 1 ). He was an astute clinician, inquisitive researcher, an extraordinarily gifted surgeon, and, above all, an excellent teacher who inspired generations of students and plastic surgeons. I had the good fortune to be under his tutelage both as a trainee surgeon and as a faculty colleague.

**Fig. 1:**
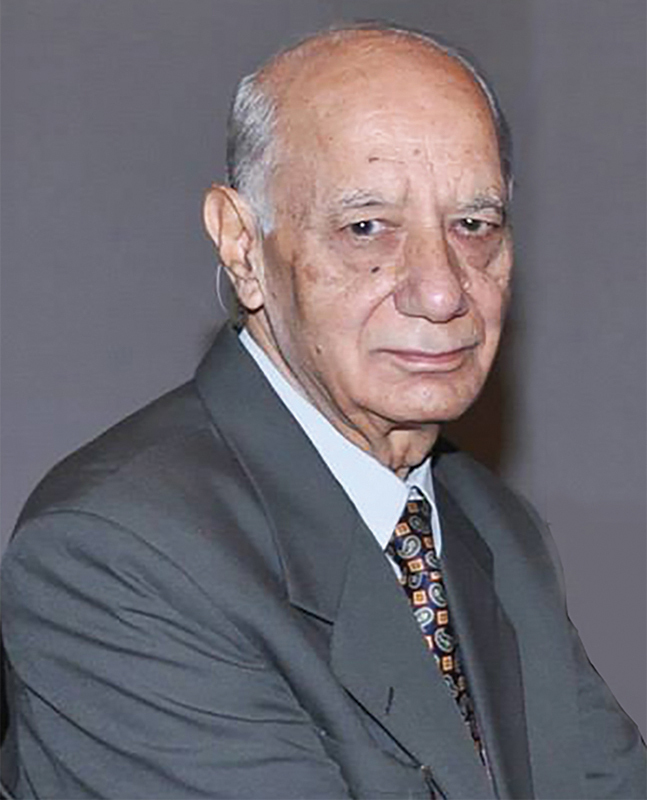
Prof. C.P. Sawhney, photographed at the Association of Plastic Surgeons of India (APSI) meeting in 2014.

Dr. Sawhney was born in Montgomery on May 15, 1930 (now in Pakistan). The family shifted to Amritsar in 1945 in view of better educational opportunities. He studied at Hindu College and later joined Glancy Medical College, Amritsar, to complete his MBBS degree. Dr. Sawhney excelled in his studies from school through postgraduation, consistently ranking as the class topper. He was resolute in his desire to become a surgeon, declining opportunities to explore other branches of medical science, despite encouragement from professors. He completed his MS General Surgery under Prof. S.S. Anand in 1956. He worked at Patiala Medical College for some time before shifting to Chandigarh.

## The Beginning of the Glorious PGIMER Journey

**Fig. 2:**
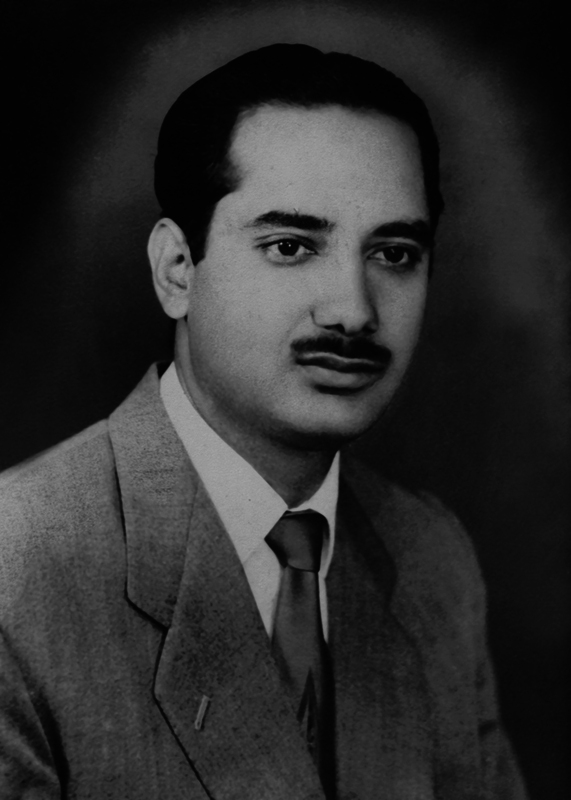
The young Dr. Sawhney at PGIMER in 1962.

Dr Sawhney joined PGIMER (Postgraduate Institute of Medical Education and Research) Chandigarh (then under Panjab government) in 1962 as a lecturer in the Department of Surgery ( Fig. 2 ). His passion for plastic surgery led him to be selected for a prestigious 1-year fellowship program in plastic surgery in the United States from 1964 to 1965. At the conclusion of the fellowship, Dr. David Phili, the program director, acknowledged that Dr. Sawhney's visit was not merely to learn but also to teach: “In this case, the examinee knew more than the examiners and has helped us learn a great deal from him.”

**Fig. 3:**
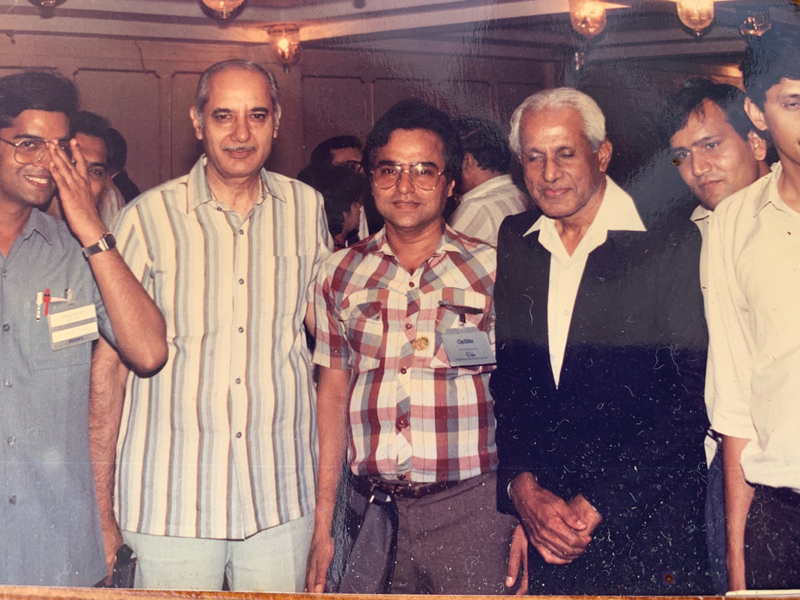
With Prof. C. Balakrishnan and Ashok Govilla in 1987.

On his return to PGIMER Chandigarh, he was given an independent unit in the newly established Department of Plastic Surgery. By this time, PGIMER Chandigarh had become a central government institution and Prof. C. Balakrishnan (CBK) had joined as professor and head of the department. These two great personalities, albeit with contrasting styles, together wrote the golden chapter of plastic surgery teaching and training in India ( Fig. 3 ). The department at PGIMER Chandigarh became the most sought-after place for any budding plastic surgeon.

## My Residency Days

**Fig. 4:**
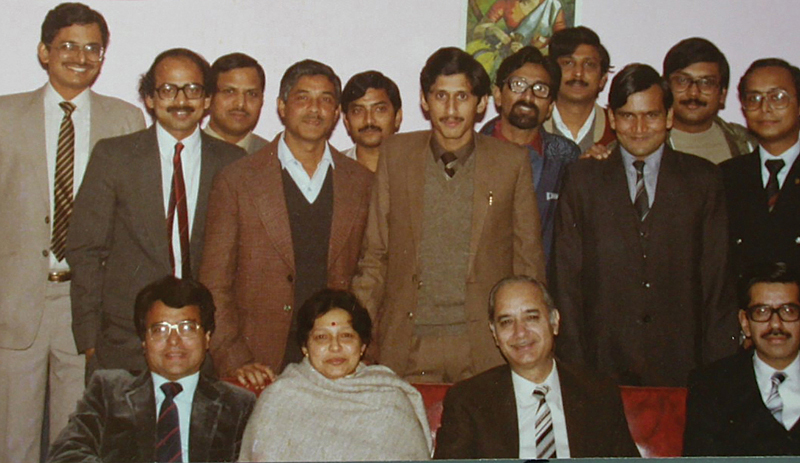
A night out at the PGIMER club: departmental get-together at the PGIMER club in 1986.

**Fig. 5:**
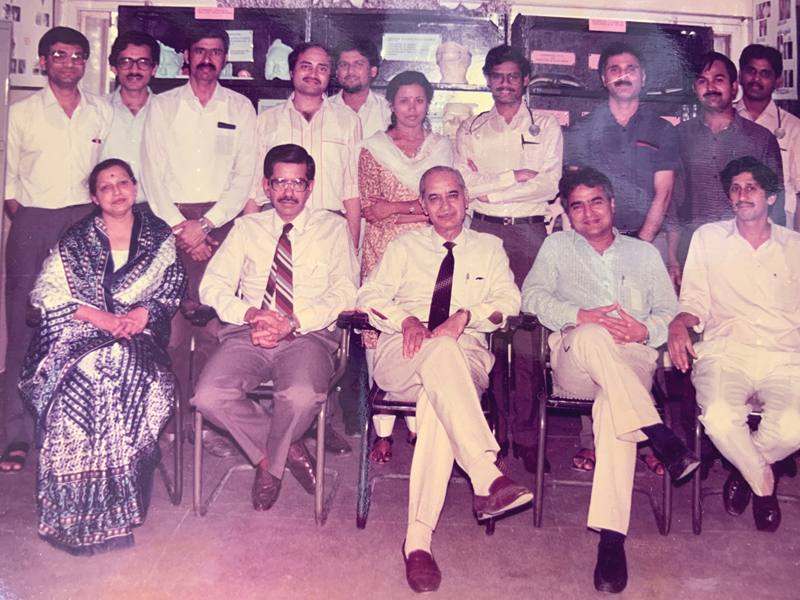
Faculty and residents, 1987.

I joined as a first-year resident in surgery at PGIMER in January 1980, and my first posting was in the Department of Plastic Surgery. I was lucky to have seen both Prof. Sawhney and Prof. Balakrishnan together in the department. I was hugely impressed by the interactions between the two stalwarts. The suave and dapper CPS always stood out because of his rational approach and affability. This admiration and respect grew further during my second rotation in the department as a third-year Junior Resident. I had all along secretly desired to be a part of the department. Fortunately, I joined the department in January 1983 to pursue MCh in Plastic Surgery. The next 7 years under his tutelage, initially as a trainee resident and later as a faculty, were defining moments in my surgical career ( Figs. 4 and 5 ).

**Innovations, Research, and Contributions:** He was an accomplished researcher and was always looking for answers to problems that were peculiar to India. His contributions to reputed British and American journals earned him international attention. He made significant contributions toward understanding the pathological anatomy of cleft lip deformity. His modification of the triangular flap repair for cleft lip defects is internationally accepted. 1 Dr. Millard pays a glowing tribute to his technique in the book *Cleft Craft* . Dr. Sawhney also made a significant contribution to the management of temporomandibular joint ankylosis. 2 He suggested a clinicopathological classification and rationale for the management of this crippling disease. He had a great interest in the management of burns patients. 3 He was the first to publish a well-conducted research paper and a clinical trial on the use of amniotic membrane in superficial burns. 4 His philosophy and techniques for the management of postburn contractures and deformities were based upon sound knowledge and practical solutions suited to the Indian scenario. He was probably the first surgeon to try primary excision in burns in India as early as 1983. He was very innovative and designed many indigenous contraptions for various situations; these include a neck splint with an extra extension of foam and an innovative axillary splint that avoided keeping the axilla in extension after axillary contracture release. 5 His innovations regarding local flap transfers in stiff areas of the scalp are also noteworthy. 6

He was a master craftsman. This was particularly obvious in nasal reconstruction performed for subtotal nasal loss. He was invited to write a chapter in the *Clinics in Plastic Surgery* . 7 He described modifications of the midline forehead flap technique with vivid details of planning, inset, and primary closure of the donor site. 8 The postoperative results of this technique were always outstanding. It was indeed a treat to watch him perform surgery. The tissue handling was delicate, the planes of surgical dissection were precise, and there was hardly any bleeding.

## The “Treatment Planning” Sessions

Prof. Sawhney had a wonderful three-dimensional visualization of the likely defect and would plan out a treatment strategy that would suit the situation perfectly. This trait was most obvious in the treatment planning sessions that we used to have every Saturday morning for 1 hour. Each of us will be given an opportunity to plan a treatment option in a case scenario. He would moderate the session asking probing questions and would always manage to elicit answers from the students. After a thorough brainstorming session, a treatment plan will be finalized, which would then be perfectly executed in the operation theater the next day. This exercise in planning was probably the best teaching session of the whole teaching program. The teaching rounds were an exercise in rational thinking. He was ready to listen to another point of view and would complement if the suggestion was correct.

He had a beautiful handwriting and good drawing skills. All his publications are full of illustrative diagrams enhancing the understanding of the message. He worked hard to establish a plastic surgery museum in the department. It housed various plaster of Paris (POP) and dental models, dissection specimens, and pictorial details of common plastic surgery procedures. The publications by the department faculty and common plastic surgery textbooks were also kept for ready reference. A facility was created in the Experimental Block of the institute to run a laboratory for learning suturing techniques on models/animals. This was also very handy in imparting initial microsurgical exposure.

## Association of Plastic Surgeons of India Honors

Prof. Sawhney was conferred many awards, orations, and medals. These include the Peet Prize for the best research paper; Ethicon Oration of the Burns Association of India; MS Verma Oration of BHU, Varanasi; Gillies' Oration of the Association of Plastic Surgeons of India (APSI) in 1987; Murari Mukherjee Oration of APSI 1995; Sushruta Oration of APSI 2002; and Founder's Oration of the Association of Cleft Lip Palate and Craniofacial Anomalies, 2004. He was invited as a visiting professor to various facilities across the globe including the United Kingdom, China, and Oman.

**Fig. 6:**
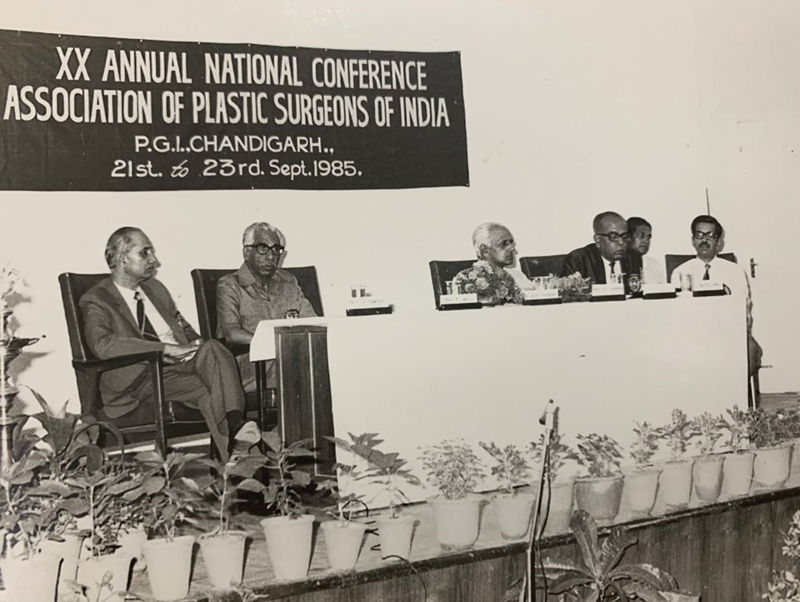
Inaugural ceremony of the Annual Conference of the Association of Plastic Surgeons of India (APSICON) 1985, Chandigarh.

**Fig. 7:**
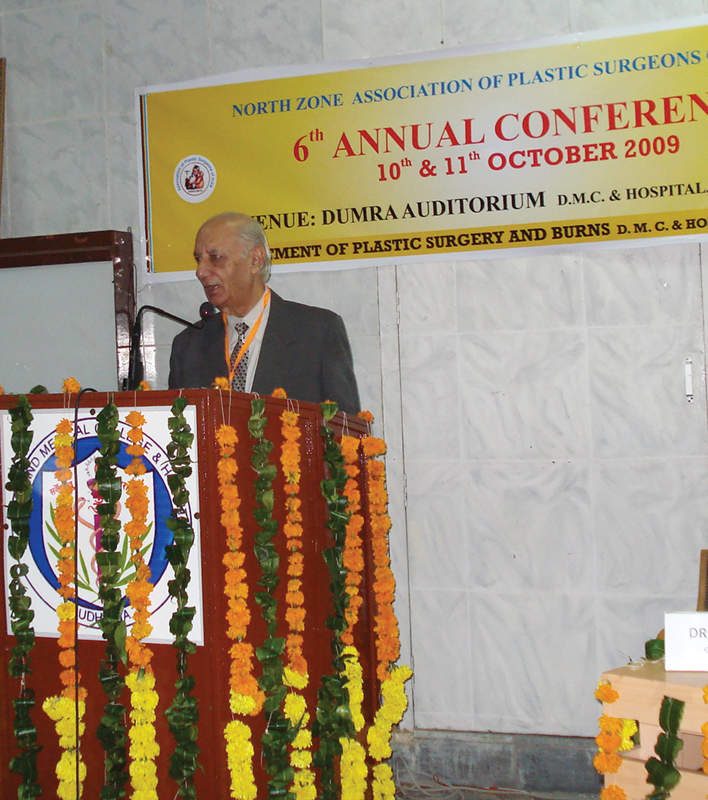
At the annual conference of North Zone Association of Plastic Surgeons of India (APSI), 2009.

He had excellent organizational skills too. Most of us fondly remember the academic and gastronomic excellence of the XX Annual APSI conference conducted at PGIMER, Chandigarh, in 1985 when he was the President of APSI ( Fig. 6 ). He was very regular in various plastic surgery meetings until he was approximately 85 years old ( Fig. 7 , 8 , 9 ). He would sit through the sessions and would be very attentive and inquisitive about the proceedings.

**Fig. 8:**
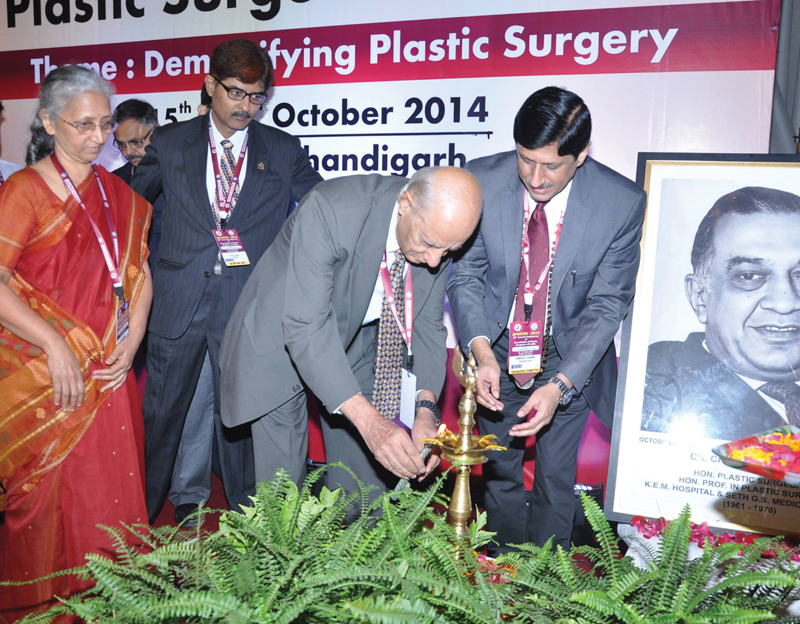
Inaugural function of the Annual Conference of the Association of Plastic Surgeons of India held in Chandigarh.

**Fig. 9:**
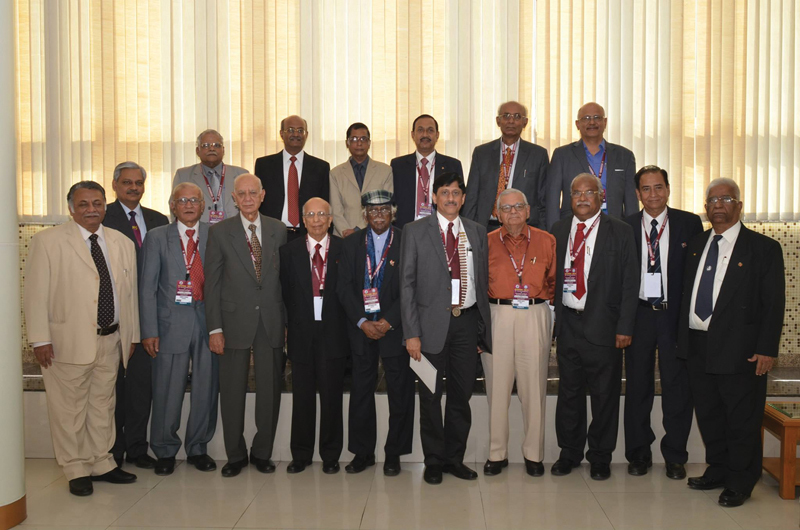
With past presidents of Association of Plastic Surgeons of India (APSI) in Chandigarh in 2014.

## Loving Family

**Fig. 10:**
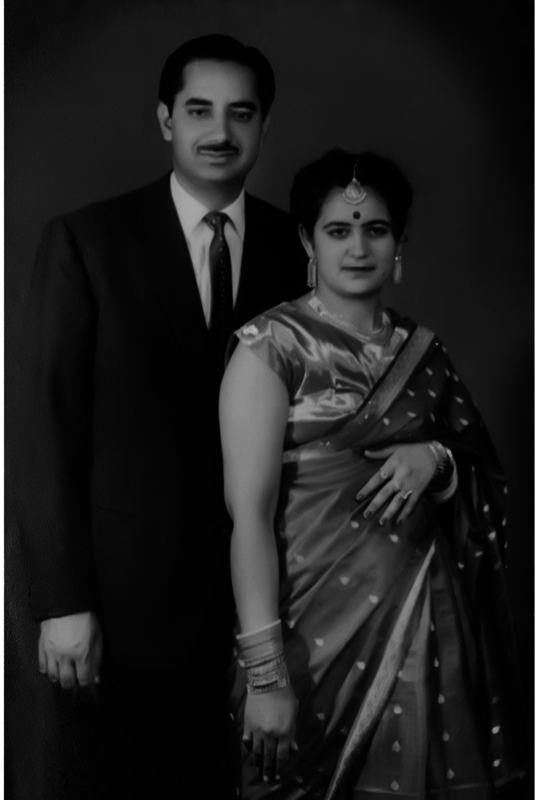
Picture perfect: with his wife Smt. Adarsh.

Dr. C.P. Sawhney was a deeply loving and caring husband, an affectionate and responsible father to his children, Mukta, Radhika, and Manish, and a doting grandfather to his granddaughters, Sanchi and Sanya. He was also a kind and loving father to his son-in-law, Rajiv, and a devoted son to his parents. His wife, madam Adarsh, was the real strength behind him ( Fig. 10 ). Dr. Sawhney frequently organized departmental get-togethers to foster camaraderie. His wife, Adarsh madam, was always a gracious host. His wife passed away in 2021.

**Fig. 11:**
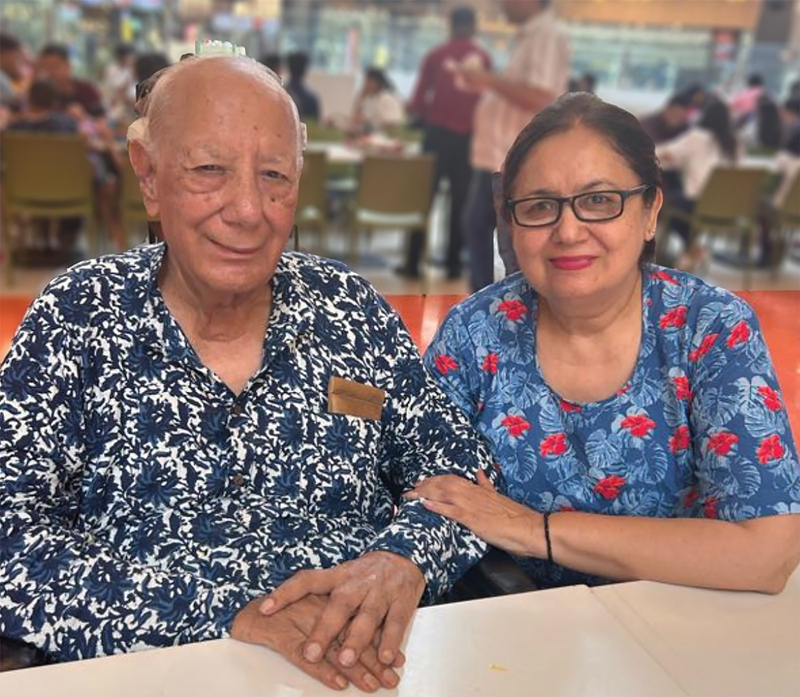
Dining out for a pizza bite with his daughter Mukta on September 11, 2024, a day before his death.

Just a week before his last breath, he visited Chandigarh and Amritsar Medical College with his loving daughter, Mukta. Dr. Sawhney embraced life with full enthusiasm. He was very fond of fast food and, in fact, went to a pizza joint just a day before his death ( Fig. 11 ).

## Empathetic Caregiver

Dr. Sawhney was a soft-spoken person who never raised his voice. The patients loved him for his kindness and empathy. His dedication to his profession and sense of duty were truly remarkable. His protective nature, especially toward women, was evident when he chose to leave his mother's cremation to attend to a young girl patient who had suffered burns because of dowry demands. One of the senior doctors who had sustained burns and was treated by him at PGIMER recalls:

“ *Deeply saddened to know about the demise of Prof. C.P. Sawhney. He saved my life. He saved me so much from being disfigured. An angel in human form. Soft-spoken, gentle, and so caring. Excellent professional expertise. I then understood the value of a good doctor–patient relationship. I used to wait for his rounds and even few minutes of his visit kept me going for the day. I still remember his gentle voice in the OT and how patiently he dressed my wounds…* .”

## “PGIMER-Sawhney Outstanding Teacher of the Year Award”

**Fig. 12:**
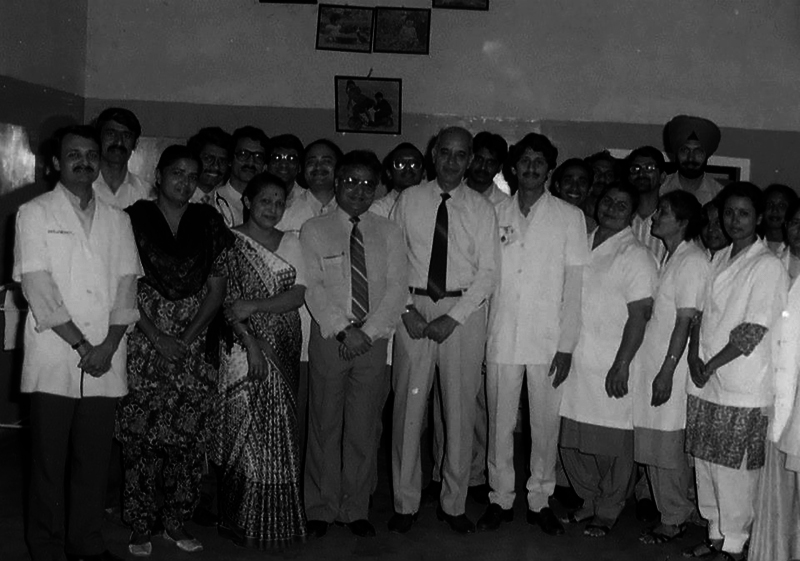
Faculty, residents, and ward staff, 1988.

He trained approximately 80 plastic surgeons under his direct supervision. Many of his trainees rose to become famous personalities nationally and internationally. Many students later became heads of academic units and proudly carried forward his legacy ( Fig. 12 ). The APSI has instituted an award entitled “PGIMER- C P Sawhney Teacher of the Year Award,” which includes a certificate, plaque, and a cash prize.

Dr. Sawhney lived a disciplined life characterized by integrity, honesty, and hard work, fully dedicated to his profession, which he cherished above all else. He embodied high thinking and simple living, believing in the principle of giving without expecting anything in return. This conviction was central to his philosophy for a happy and contented life, which he practiced until his final days.

He was not only a great plastic surgeon and an inquisitive researcher but also an extraordinary human being and an amazingly effective motivator. He truly dedicated his life to advancing plastic surgery, educating generations and hundreds of plastic surgeons across the world.

He was a man who walked the talk—one who preached what he practiced.

**Fig. 13:**
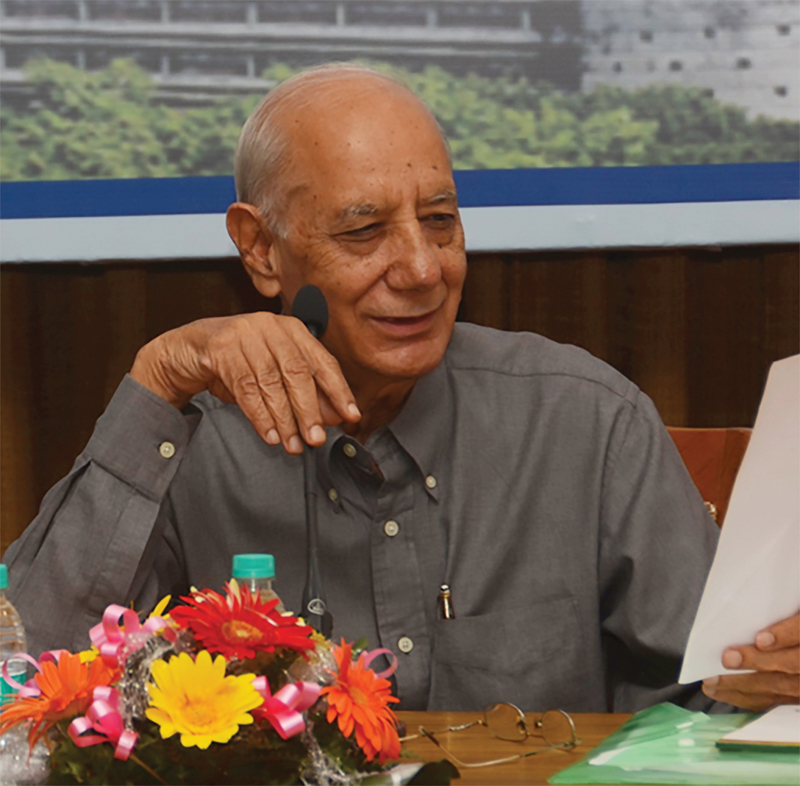
The affable boss wielding the mike at the Continuing Medical Education (CME) in 2012 in Chandigarh.

Such great persons never die; they continue to live and spread happiness through good work. His innumerable students and admirers would ensure that his legacy continues ( Fig. 13 ).

This is our humble tribute to this wonderful, multifaceted personality.
